# Modulating Mesothelin Shedding to Improve Therapy

**DOI:** 10.18632/oncotarget.445

**Published:** 2012-02-09

**Authors:** Yujian Zhang, Ira Pastan

**Affiliations:** ^1^ The Laboratory of Molecular Biology, Center for Cancer Research, National Cancer Institute, National Institutes of Health, Bethesda, Maryland, USA

Many tumor-specific or tumor-associated antigens have been discovered, but only a few of them have been successfully used for cancer therapy. Not only are the pharmacological properties of therapeutic drug molecules critical to the clinical success, the biological nature of the target antigens has a big influence on the therapeutic outcome.

In searching for good drug targets for cancer, our lab discovered a cell surface protein called mesothelin in the 1990s. It is highly expressed in many tumors such as mesothelioma, ovarian cancer, pancreatic cancer and lung cancer. However, it only has minor expression in normal mesothelial cells lining the pleura, pericardium and peritoneum. This expression pattern makes it an attractive target. We designed a mesothelin targeted immunotoxin called SS1P in which the Fv portion of an anti-mesothelin antibody is fused to a truncated portion of *Pseudomonas* exotoxin A (PE38).

The cell-killing mechanism of immunotoxin involves three general steps. i) The SS1P immunotoxin molecule binds to mesothelin on the cell surface; ii) SS1P enters the cell through a mesothelin-mediated internalization process and its PE portion is translocated to the cytosol; and iii) the PE molecule causes ADP-ribosylation of EF-2, leading to arrest of protein synthesis and apoptosis. SS1P turns out to be very toxic to mesothelioma cells, yet is well tolerated by patients in spite of low level mesothelin expression in normal tissue. We observed several minor responses in our phase I clinical trials [1].

Depending on the cell-killing mechanism, the cytotoxicity of an immunotoxin can be significantly affected by the target properties, such as the signaling pathway involved, the cell surface target density, target internalization rate, target-mediated membrane traffic pathway, etc. Mesothelin is a GPI-anchored protein without an intracellular tail. Its physiological ligand has not been identified and it is not known if mesothelin is involved in any signaling pathway. Mesothelin has been shown to bind to another cell surface protein, MUC16.

Mesothelin has several features, which make it useful for cancer therapy. Mesothelin is well-internalized, making it a good target for immunotoxins. Mesothelin is actively shed from the cell surface, generating an antigen pool in the circulation and in the tumor interstitial space. The measurement of serum levels of shed mesothelin is used as a criterion in the diagnosis of mesothelioma and ovarian cancer in patients. Mesothelin in the tumor interstitial space will inevitably interact with its targeting agents during the process of tumor penetration. To better understand the biological and pharmacological significance of mesothelin shedding, we studied the mechanism of shedding in the A431/H9 cell line, which highly expresses mesothelin on its cell surface [2]. Although mesothelin is attached to the cell membrane through its GPI anchor and phospholipase (PL) can release the protein, PL does not seem to play a significant role in the shedding because several PL inhibitors failed to reduce the shedding, and sequencing of shed mesothelin did not reveal the amino acid sequence expected from PL C action [2, 3]. Instead, we found that broad-spectrum MMP/ADAM inhibitors significantly inhibit mesothelin shedding. We narrowed down the candidate MMP/ADAM genes by testing the inhibitory effect of several endogenous MMP/ADAM inhibitors. These studies led us to ADAM17/TACE, the first ‘sheddase’ to be identified, which has been implicated in the release of a diverse variety of membrane-anchored cytokines, cell adhesion molecules, receptors, ligands and enzymes. TACE knockdown by siRNA significantly reduced mesothelin shedding.

TACE is a transmembrane glycoprotein, most noted in cancer for its role in releasing EGFR ligands from the cell surface, by which it can regulate the activation of the EGFR pathway. There is evidence that chemotherapy can activate TACE, contributing to drug resistance, whereas inhibiting TACE can sensitize tumor cells to chemotherapy [4, 5]. We observed that cytotoxicity of SS1P was increased in cultured cells when TACE was knocked down by siRNA or inhibited by small inhibitor molecules. We do not know whether the EGFR pathway plays a role in this finding but we saw increased cell surface mesothelin levels and an increased internalization rate of SS1P. These are favorable characteristics for SS1P therapy. Considering the possibility of translating our research finding to the clinic, we examined other mesothelin-expressing cell lines and primary mesothelioma cells from several patients. TACE inhibition was shown to sensitize these cells to SS1P. We attribute this effect to the reduced dissociation of targeting molecules bound to mesothelin from the cell surface after binding. Currently there is no effective ADAM17 inhibitor available for clinical studies. Several broad-spectrum MMP/ADAM inhibitors failed in clinical trials, so it would probably be difficult to get a protocol approved to directly evaluate this approach in patients. There is a new small molecule called INCB7839 which is showing some promising results [6].

We want to emphasize that tumor targeting is a complicated process. The modulation of mesothelin shedding could have a systemic influence on drug kinetics in both circulation and tumor tissue. Our *in vitro* assay did not take this into consideration, which could be essential when developing a therapeutic strategy. Besides shedding, mesothelin levels could be modulated like other antigens by trogocytosis [7] or antigen masking [8]. Whether they play a role in mesothelin targeted therapy remains to be evaluated. An understanding of possible mesothelin modulation will be instrumental to new targeting strategies.
